# Book Reviews

**DOI:** 10.1155/S1463924679000316

**Published:** 1979


### Book Reviews

### Evaluation and optimisation of laboratory methods and analytical procedures

**By D. L. Massart, A. Dijkstra and L.Kaufman**

*1978 Elsevier Scientific Publishing Company, Amsterdam, Oxford, New York. pp.596 $57.75 ISBN 0-444-41743-5.*

This is a book of value to both the analytical chemist and the statistician, in particular to the statistician who is working in the field of analytical chemistry. For the analytical chemist to obtain maximum value from the book he will need a greater knowledge of statistical techniques than is normally obtained from the average undergraduate chemistry course. The book is divided into five parts and throughout it examples are given of the application of the optimisation techniques to analytical methods.

*Part I: Evaluation of the Performance of Analytical Procedures.* This section gives many definitions and points out some discrepancies between those commonly used. It introduces statistical methods indicating their use. It gives details of formulae but these are generally kept together at the end of the chapter under the heading 'Mathematical Section' (this system is maintained throughout the book). There are chapters covering the following topics: performance characteristics, precision and accuracy applied to more than one set of data, reliability and drift (although I should like to have seen more on Analytical Quality Control), sensitivity and limit of detection (this chapter is confusing in places and could possibly have been taken a little further), selectivity and specificity, and the practicability and costs of techniques. The whole of this section of the book would be extremely valuable to analytical chemists.

*Part II: Experimental Optimisation.* This section begins with a survey of experimental optimisation methods followed by detailed chapters on Factorial analysis and Sequential experimental designs covering Simplex methods. The final chapter examines Steepest Ascent and Regression methods. The whole of this very valuable Part would repay careful reading.

*Part III: Combinational Problems.* As in the previous section it begins by giving an introduction with examples of problems that may be encountered within the Laboratory, it then goes on to deal with the solutions. The topics covered are: the Multivariate methods of Cluster, Factor, Principle Component and Discriminant Analysis, Operational Research Methods of Linear programming, Queueing theory and Graph theory and related topics. This might be the most difficult part of the book for the average analytical chemist to understand.

*Part IV: Requirements for Analytical Procedures.* This section deals with analytical errors, process monitoring and control. These chapters are well worthwhile reading as they cover subjects that are frequently neglected.

*Part V: Systems Approach in Analytical Chemistry.* This gives a systems analysis of the practice of analytical chemistry, breaking down the procedures into the various component parts. The final chapter gives an account of the organisation of an Analytical Laboratory.

This is a good book, it is well written and fairly easy to understand. The book is biased towards the practical application of the techniques described. It brings together in the one book a considerable amount of useful information. Each chapter concludes with a comprehensive list of up to date references mostly taken from journals in the field of analytical chemistry. It would be of particular value to the analytical chemist who is prepared to put some effort into acquiring a knowledge of these techniques, and to the statistician who requires information on the practical application of these techniques.

The book appears to have been produced by offset litho from a typescript but the quality of the typeface is very good and the book is easy to read unlike many publications produced by this technique.

*J. Vernon*

### Compendium of Analytical Nomenclature

**Edited by H. M. N. H. Irving, H. Freiser, T. S. West.**

*1978, Pergamon Press Ltd., Oxford, pp228, £14.00 (hardback), £8.00 (softback). ISBN 008-0220088 (hardback), 008-0223478 (softback).*

For the last 20 years the Analytical Division of the International Union of Pure & Applied Chemistry (IUPAC) has had the unenviable task of obtaining world-wide consensus on the standardisation of nomenclature and symbols for use in analytical chemistry. Individual preliminary reports have been published in *Pure & Applied Chemistry* from time to time and at least 8 months have been allowed subsequently for comment to be received. After this was taken into account the definitive versions of the reports were produced and have now been gathered together in the compendium.

Previous comparable IUPAC compendia have included coverage of the symbols and nomenclature of organic and inorganic chemistry, physical quantities and units, etc., none of these either attempted or needed to cover the variety of topics which fall under the heading of 'analysis'. In the preamble to the book it is stressed that the membership of the various commissions responsible for the work within the Analytical Division, has changed considerably over the last 20 years and this, plus the fact that different commissions approached their problems in different ways, has meant that there is a lack of consistency in the approach adopted to the various chapters. Precise definitions in some cases give way to discursive prose treatment of the meanings of various terms and though this makes for interesting reading, for reference purposes, required data in various areas may be somewhat difficult to find. There are 21 short chapters whose coverage ranges from general recommendations on the reporting of results (with definitions of accuracy, precision, etc.) to recommendations for specific types of instruments and systems. Appendix deals with the usage of the terms 'equivalent' and 'normal'.

*F. L. Mitchell*

### Computers in Mass Spectrometry

**by J. R. Chapman**

*1978 Academic Press, London. pp.275, £9.80. ISBN 0-12-168-7503.*

Review texts on the use of computers in mass spectrometry are scarce and publication of this book should be welcome to most laboratories, workers and students in the field of organic mass spectrometry. The reasons why few books have been attempted on this topic perhaps lie in the rapidly changing nature and diversity of computer hardware, and the difficulty in treating the subject in a way which would not limit its readership to computer experts, but also be of value to the general reader. The first of these problems the author has avoided by strictly limiting his description of mass spectrometric and computer hardware on the grounds that any detailed discussion would soon be out of date. On balance this seems a wise decision. A comprehensive number of aspects of the topic are covered in the book at a fairly high level but at one which will require the reader to refer to original papers on individual aspects for more detailed information. This approach would seem essential to restrict over-emphasis of specialised detail which has limited interest, and maintain the objective of the book which is an overview of computer implementation in mass spectrometry.

The chapters fall naturally into two parts. In the first four chapters the author deals with instrumentation and data acquisition, conversion and reduction, while the remaining chapters include approaches to the interpretation and treatment of this data with a final chapter on quantitative analysis.

Fashions in the most acceptable computer system for a laboratory have changed since they were first introduced into mass spectrometry, passing through the stage when centralised computers were advocated.

The author discusses the merits of different arrangements but uses the on-line dedicated mini-computer as the model system when considering acquisition of data. Such systems are the most commonly adopted at present and are likely to remain so with improving specifications, although microprocessors will undoubtedly influence aspects of design.

The excellent chapter on library search should be of value to many workers interested in finding suitable routines to cope with their own individual needs for library facilities. Obviously no single programme is satisfactory for all problems, and the chapter sets out well the various ways of compiling library files of mass spectra and the merits of different systems for searching these files to match an unknown spectrum. When the library file does not contain the unknown spectrum then there is a need for the library programmes to pick out structurally similar compounds or to go further and attempt to elucidate the structural features from known fragmentation rules and other information. Interpretative programmes are extremely large and the author has conveniently summarised the principles involved. In addition the complexities of pattern recognition algorithms applied to unknown spectra should help most readers to follow the expanding literature in this area.

The control of several parts of the mass spectrometer and the acquisition and processing of data from quantitative analysis can be carried out by computer for reasons of convenience, improved precision and the measurement of often large numbers of samples of the same type. The author has reviewed developments in this area in a way, like most of the text, which will give the reader a broad understanding of the progress and utility of computer application. For this reason this book is a worthwhile addition to the bookshelves of all users of mass spectrometers and data systems.

*A. H. Lawson*

### New Literature

### Pharmaceutical sciences

Technicon Industrial Systems has published a special issue of the Pharmaceutical Sciences Newsletter, of particular interest to methods development laboratories now involved with HPLC technology and with the fast-approaching USPXX dissolution requirements. The special newsletter issue features an article on the analytical and physical chemistry section of Wyeth Laboratories Inc., Radnor, a long-term user of continuous-flow systems. Faced with the need to speed up HPLC assays, Wyeth successfully automated a stability-indicating assay for benzodiazepines, using a fully automated analytical system combining sample treatment with liquid chromatography separation techniques.

Copies of the newsletter can be obtained by writing to Technicon Industrial Systems, Tarrytown, New York 10591, U.S.A.

### Photometer for amino acid analysis

A two-page data sheet is now available for the Dionex Model D-550 Dual Channel Ratio Photometer, a high performance detector for amino acid analysis. The data sheet lists features of the photometer and describes the principles of operation. In addition, a functional diagram illustrates the photometer and its relationship to an amino acid analyser. Also included is a table of applications, a figure showing dynamic range and a list of specifications. The Model D-550 employs a number of novel techniques to expand the linear absorbance range to 3.5 Abs and improve the signal-to-noise ratio.

Copies of the sheet are available from Dionex Corporation, 1228 Titan Way, Sunnyvale, CA 94086, U.S.A.

### Water analysis

The Ionics range of laboratory monitors, the 1200 series, for the analysis of natural and waste waters, available from Techmation, are described in a 6-page booklet available on request. The series includes total oxygen demand, total carbon, total organic carbon and combined total oxygen demand/total organic carbon analysers. All the analysers feature an all ceramic sample injection valve that provides accuracy and a repeatable performance with low maintenance. They are fully automated and simple to operate. Reliable solid state electronics give automatic zeroing prior to each analysis and provide precisely controlled operations. The 1200 series can be interfaced with an auto-sampler: the operator is able to analyse and record the results of up to 200 individual samples.

The booklet is available on request from Techmation Ltd., 58 Edgware Way, Middx., U.K.

### High performance thin-layer chromatography

A 16-page CAMAG bulletin 'HPTLC' contains their new HPTLC developing system, several variations of the circular developing device 'U-Chamber' acc. to R. E. Kaiser, and two quantitative submicroliter sample applicators.

A separate 4-page bulletin presents a new TLC/HPTLC scanner, a photodensitometer that has been designed to scan automatically all tracks of a chromatogram, be it conventional or high-performance TLC, developed linear or circular.

Both bulletins are available free from CAMAG, Homburgerstrasse 24, CH-4132 Muttenz/Schweiz.

### Semiconductor devices

Data I/O have recently published a 32-page booklet entitled 'How to survive in the bipolar PROM, FPLA, PAL, MOS EPROM, diode matrix, PMUX, and gate array Programming Jungle.' The book is a good introduction to the many aspects of programmable semiconductor devices and programming techniques.

The first major section of the book discusses the various semiconductor technologies that are used to produce programmable devices. This is followed by tips on handling MOS devices and a method to assess the effectiveness of the ultra-violet lamp used to erase MOS EPROMs. Having provided the reader with the basic background knowledge, the booklet goes on to explain how the devices are programmed under both manual and automatic computer control. Each process is covered and all the jargon normally found in literature describing programming equipment is explained in simple terms. A section is included which provides details of the range of Data I/O programming machines. These machines cover all programming needs from simple 'one off' programming to batch programming on a production line basis. The final pages of the book list the key features of about 300 programmable

